# Multi-Parametric Ultrasound Radiomic Kinetics with Machine Learning Ensemble for Early Prediction of Pathologic Response to Neoadjuvant Chemotherapy in Breast Cancer

**DOI:** 10.3390/diagnostics16162504

**Published:** 2026-08-08

**Authors:** Ramona Putin, Livia Stanga, Ciprian Ilie Roșca, Horia Silviu Branea, Adrian Cosmin Ilie, Alina Tanase, Coralia Cotoraci

**Affiliations:** 1 Doctoral School, Faculty of Medicine, Vasile Goldis Western University of Arad, 310414 Arad, Romania; ramonaputin@yahoo.com (R.P.); cotoraci.coralia@uvvg.ro (C.C.); 2 Discipline of Microbiology, Faculty of Medicine, “Victor Babeș” University of Medicine and Pharmacy, 300041 Timisoara, Romania; stanga.livia@umft.ro; 3 Department of Internal Medicine I—Medical Semiotics II, “Victor Babeș” University of Medicine and Pharmacy, 300041 Timisoara, Romania; 4 Department of Functional Sciences, Discipline of Public Health, Center for Translational Research and Systems Medicine, “Victor Babeș” University of Medicine and Pharmacy, 300041 Timisoara, Romania; ilie.adrian@umft.ro; 5 Department of Professional Legislation in Dental Medicine, Faculty of Dental Medicine, “Victor Babeș” University of Medicine and Pharmacy, Eftimie Murgu Square No. 2, 300041 Timisoara, Romania; tanase.alina@umft.ro; 6 Research Centre in Dental Medicine Using Conventional and Alternative Technologies, Faculty of Dental Medicine, “Victor Babeș” University of Medicine and Pharmacy, Eftimie Murgu Square 2, 300041 Timisoara, Romania

**Keywords:** breast neoplasms, neoadjuvant therapy, elasticity imaging techniques, ultrasonography, machine learning

## Abstract

**Background/Objectives**: Early identification of breast cancer patients unlikely to benefit from neoadjuvant chemotherapy (NAC) remains a pressing clinical problem because ineffective therapy delays definitive surgery and exposes patients to unnecessary toxicity. Quantitative ultrasound (QUS) and shear wave elastography (SWE) probe complementary tissue properties—scatterer microstructure and mechanical stiffness—that may change before macroscopic tumor shrinkage. This study aimed to evaluate whether multi-parametric ultrasound (mpUS) radiomic kinetics, analyzed with a machine learning ensemble and interpreted with SHAP, could predict pathologic response to NAC. **Methods**: A prospective observational cohort enrolled 135 women with biopsy-proven stage II–III breast cancer treated with NAC within the multidisciplinary breast pathway shared between Vasile Goldis Western University of Arad and Victor Babes University of Medicine and Pharmacy Timisoara (Pius Brinzeu County Emergency Hospital). All patients underwent standardized QUS and SWE acquisitions at baseline, week 1, and week 3. Response was defined pathologically at surgery as residual cancer burden (RCB) class 0/I versus II/III. Group comparisons used Welch’s *t*-test, Mann–Whitney U, chi-square, and Fisher’s exact tests; correlations used Spearman’s rho. A stacked machine learning ensemble (four base learners—XGBoost, random forest, support vector machine, and L2-penalized logistic regression—combined by a separate second-stage logistic meta-learner) was trained with nested 10-fold cross-validation, bootstrap stability assessment, SHAP-based interpretability, and decision curve analysis. **Results**: Sixty patients (44.4%) were responders and 75 (55.6%) were non-responders. Responders showed greater week 3 increases in mid-band fit (3.4 ± 0.9 vs. 1.2 ± 0.8 dB, *p* < 0.001), entropy (0.7 ± 0.2 vs. 0.2 ± 0.2, *p* < 0.001), and more pronounced SWE mean stiffness reduction (−44.1 ± 9.7 vs. −12.1 ± 8.6 kPa, *p* < 0.001). The stacked ensemble integrating clinical, QUS, and SWE kinetic features reached an AUC of 0.93 (95% CI 0.88–0.97) versus 0.71 for the clinical-only model (all reported performance figures represent internal cross-validation only). SHAP analysis identified Δ MBF and Δ entropy at week 3 as the dominant features, with high bootstrap stability. **Conclusions**: Multi-parametric ultrasound radiomic kinetics integrated through a machine learning ensemble may provide an interpretable early-response biomarker for NAC in breast cancer, pending external validation.

## 1. Introduction

Breast cancer remains the most frequently diagnosed malignancy in women worldwide, with persistent variation in mortality and access to multimodal care across regions [1]. Neoadjuvant chemotherapy (NAC) has become a standard component of care for locally advanced and biologically aggressive disease because it can downstage the tumor, increase the rate of breast-conserving surgery, and function as an in vivo test of treatment sensitivity, with residual disease at surgery providing strong prognostic information [2]. The challenge that persists at the bedside is to determine, during the early weeks of treatment, whether the regimen is producing a meaningful tissue-level effect or whether the patient is being exposed to cytotoxicity without proportionate benefit. NAC is considered standard treatment for locally advanced and biologically aggressive breast cancer because downstaging the tumor converts many patients who would otherwise require mastectomy into candidates for breast-conserving surgery, and because the pathologic response at surgery—particularly the residual cancer burden—is a validated surrogate that stratifies long-term event-free and overall survival [2,3].

Current response monitoring during NAC is dominated by clinical examination and serial morphologic imaging, principally ultrasound and magnetic resonance imaging, alongside the surgical pathology endpoint of residual cancer burden [3,4]. Tumor size reduction remains clinically valuable, but macroscopic shrinkage often lags behind cellular and microstructural changes induced by chemotherapy, and Ki-67 reduction on interval core biopsy remains invasive and inconsistently performed. MRI offers excellent soft-tissue contrast and is the imaging reference standard, yet it is expensive, time-consuming, less tolerated in serial follow-up, and unsuitable for very frequent reassessment within the first treatment cycles [5]. The core difficulty of the early treatment weeks is therefore that macroscopic tumor-size reduction is an insensitive early readout, because cellular and microstructural changes induced by chemotherapy typically precede measurable shrinkage; MRI, despite the soft-tissue reference standard by virtue of its high contrast resolution, is too costly and time-consuming for the repeated within-cycle assessment that early decision-making would require [5].

Quantitative ultrasound has emerged as an attractive complementary modality because it converts the radiofrequency information underlying conventional B-mode imaging into numerical descriptors of tissue scatterer organization [6]. Mid-band fit, spectral slope, spectral intercept, and gray-level co-occurrence features such as entropy and homogeneity capture microstructural information that is not visible to the eye on standard images. After effective chemotherapy, apoptosis, necrosis, stromal remodeling, and edema disrupt scatterer spacing and tissue regularity, producing measurable QUS shifts well before any volumetric change is clinically detectable [7]. This makes QUS a biologically plausible candidate for early response assessment. In mechanistic terms, QUS characterizes tissue at the sub-resolution scale of scatterer size, spacing, and concentration, and gray-level co-occurrence matrix (GLCM) descriptors such as entropy and homogeneity quantify the spatial regularity of these scatterers, so that chemotherapy-induced apoptosis, necrosis, stromal remodeling, and edema register as measurable increases in textural disorder [6,7].

Shear wave elastography adds a fundamentally different physical dimension to the tissue characterization, namely mechanical stiffness, measured quantitatively in kilopascals [8]. Untreated breast cancers tend to be stiff because of dense collagenous stroma and cellular packing, and effective cytotoxic therapy typically reduces stiffness through cell loss, stromal degradation, and inflammatory softening [9]. Combining QUS with SWE therefore yields a multi-parametric ultrasound (mpUS) signature in which scatterer-derived radiomic features and elasticity-derived biomechanical features describe partially independent aspects of the tumor response. To date, however, most prediction models have used these modalities separately rather than integrating them with formal kinetic modeling across multiple timepoints [10]. On shear-wave elastography, untreated invasive carcinomas are characteristically stiff owing to dense collagenous stroma and high cellular packing, whereas effective cytotoxic therapy softens the tumor through cell loss, stromal degradation, and inflammatory change, so a fall in stiffness supplies a mechanistically independent early signal that complements the scatterer-based QUS readout [8,9].

Machine learning is increasingly used to integrate high-dimensional imaging features with clinical variables, but model opacity remains a clinical barrier. Modern interpretability tools such as Shapley Additive exPlanations (SHAP) attribute the contribution of each feature to each individual prediction, transforming the model from a black box into a transparent rule-based system in which the clinician can see which features pushed the probability up or down for a given patient [11]. Ensemble approaches that stack heterogeneous base learners have repeatedly outperformed single algorithms in moderate-sized clinical datasets and can be combined with self-attention and federated-learning frameworks that improve robustness and interpretability [12,13,14]; gradient-boosted decision trees are a standard high-performing component of such ensembles [15], and bootstrap stability assessment further allows reporting of the reliability of feature rankings [16]. Combining QUS with SWE is expected to be more informative than either modality alone because scatterer-derived and elasticity-derived features describe partially independent biological axes (microstructure versus mechanics); stacked ensembles are advantageous in moderate-sized datasets because averaging heterogeneous base learners reduces variance and the risk of overfitting, and bootstrap stability assessment quantifies how reproducibly each feature retains its importance across resamples [15,16].

The present study was designed to evaluate whether a multi-parametric ultrasound radiomic kinetic profile—obtained at baseline, week 1, and week 3—integrated through a stacked machine learning ensemble and interpreted with SHAP can predict the pathologic response to NAC in a real-world breast oncology cohort. We hypothesized that responders would display a distinct baseline mpUS phenotype and far more pronounced early kinetic changes than non-responders, and that the ensemble combining clinical, QUS, and SWE kinetic features would substantially outperform clinical and single-modality baseline approaches, in line with prior reports indicating that radiomic and subtype information can be additive [17]. To our knowledge, this is the first study to integrate QUS spectral–textural radiomics and SWE elasticity as a joint, multi-timepoint kinetic signature analyzed with a stacked ensemble and SHAP; we hypothesized that responders would exhibit a distinct baseline mpUS phenotype and substantially larger early kinetic changes than non-responders, and that the combined kinetic model would outperform clinical and single-modality baselines [10,17]. The present work extends an earlier QUS-only analysis by our group, which examined baseline and early-delta spectral features at a single on-treatment timepoint in a smaller single-center cohort and did not incorporate elastography, multi-timepoint kinetic modeling, or ensemble learning [18].

## 2. Materials and Methods

### 2.1. Study Design and Clinical Setting

This investigation was structured as a prospective single-center observational cohort study performed within the multidisciplinary breast oncology pathway shared between the Doctoral School of the Faculty of Medicine, Vasile Goldis Western University of Arad, and Victor Babes University of Medicine and Pharmacy Timisoara, with all patients receiving treatment at the Pius Brinzeu County Emergency Hospital Timisoara. Recruitment took place over a continuous enrollment window from January 2024 to December 2025. A QUS-only analysis of baseline and early-delta spectral features arising from this institutional breast imaging program has been reported separately [18]; the present study is distinct in that it adds shear wave elastography, a third acquisition timepoint, and a stacked ensemble with SHAP interpretability. The institutional review board approved the protocol and written informed consent was obtained from every participant. Imaging and clinicopathologic data were collected within a single coordinated workflow to minimize technical heterogeneity, and all multi-parametric ultrasound acquisitions were performed on the same scanner platform by two operators trained with a common protocol. A prospective observational design was chosen because it captures response as it unfolds under routine care without altering treatment, and technical heterogeneity was minimized by acquiring all imaging within a single coordinated workflow on one scanner platform with locked presets, since QUS spectral parameters and SWE stiffness are sensitive to differences in transducer, transmit frequency, and signal-processing pipeline across systems; the overall study workflow and analytic pipeline are summarized in the study flowchart presented in Section 3.2.

The principal aim was to determine whether multi-parametric ultrasound radiomic kinetics could distinguish patients who would ultimately achieve a favorable pathologic response at surgery from those left with substantial residual disease and to develop an interpretable machine learning ensemble that captured this signal early in treatment. The study deliberately included three timepoints—baseline before the first chemotherapy cycle, week 1 after one cycle, and week 3 just before the second cycle—because both the very early acute tissue response and the slightly more mature consolidated response carry complementary biologic information. Imaging acquisition did not alter the clinical decisions of the multidisciplinary tumor board, and standard NAC regimens were administered without modification driven by the radiomic findings. The three timepoints were selected deliberately as follows: baseline establishes the pretreatment phenotype, week 1 captures the very early acute tissue response (apoptosis, edema, and initial stromal change) that can precede any measurable shrinkage, and week 3—after a full cycle—reflects a more consolidated response, so that week 1 and week 3 provide complementary early and maturing views of treatment effect.

### 2.2. Patient Selection and Definitions

Eligible patients were adult women with newly diagnosed, biopsy-proven, unilateral invasive breast carcinoma classified as stage II or III, with a measurable index lesion suitable for reproducible ultrasound assessment, scheduled to receive standard anthracycline- and taxane-based neoadjuvant chemotherapy with the addition of HER2-directed therapy when indicated. Inclusion required the availability of complete pretreatment clinicopathologic data, baseline mpUS acquisition before the first cycle, repeat mpUS at week 1 and week 3, definitive surgical pathology with residual cancer burden classification, and signed informed consent. The exclusion criteria comprised metastatic disease at presentation, prior ipsilateral breast surgery, prior radiotherapy or systemic therapy, pregnancy, bilateral synchronous tumors without a clearly identifiable index lesion, lesions not consistently visualized on ultrasound, incomplete or technically inadequate mpUS data at any timepoint, and missing surgical pathology. Only unilateral invasive carcinoma with a measurable, reproducibly visualized index lesion was included so that serial ROI placement and feature extraction would be reliable across timepoints; patients with metastatic disease were excluded because their systemic management and prognosis differ fundamentally from curative-intent NAC, prior ipsilateral surgery or radiotherapy were exclusions because treated tissue alters baseline scattering and stiffness, and bilateral synchronous tumors were admitted only when a single unambiguous index lesion could be designated for longitudinal measurement.

The pathologic response at surgery served as the reference standard. A favorable response was defined as residual cancer burden class 0 or I, while an unfavorable response was defined as residual cancer burden class II or III, scored at the institutional pathology laboratory according to the standardized BIG-NABCG methodology and verified by a senior breast pathologist blinded to the imaging results [19]. The molecular subtype was categorized using immunohistochemistry and HER2 status as Luminal B, HER2-positive, or triple-negative breast cancer (TNBC). Extracted clinical variables included age, body mass index, clinical TNM stage, histologic grade, longest lesion diameter, and baseline Ki-67 labeling index. Interval Ki-67 from a week 3 core biopsy was available for the entire cohort by protocol, and residual cellularity at definitive surgery was recorded for all patients. Molecular subtype was assigned from immunohistochemistry and HER2 status into Luminal B, HER2-positive, or triple-negative categories; the extracted clinical variables (age, body mass index, clinical TNM stage, histologic grade, longest diameter, and baseline Ki-67) were selected because each is an established correlate of chemosensitivity, and an interval Ki-67 at week 3 was mandated because a decline in proliferative index is an early pharmacodynamic marker of cytotoxic effect. The protocol-mandated week 3 core biopsy for interval Ki-67 was completed by all 135 enrolled patients (100% compliance); because the repeat biopsy was a prespecified inclusion requirement, patients unable or unwilling to undergo it were not enrolled rather than retained with missing data.

### 2.3. Multi-Parametric Ultrasound Acquisition and Feature Extraction

All examinations were performed on a single research-enabled ultrasound platform (Aixplorer MACH 30; SuperSonic Imagine/Hologic, Aix-en-Provence, France) equipped with a high-frequency linear-array transducer operated at 5–12 MHz and dedicated firmware allowing simultaneous capture of beamformed radiofrequency data, B-mode images, and two-dimensional shear wave elastography elastograms in the same plane and during the same patient visit. Standardized acquisition settings were applied across patients and timepoints as follows: imaging depth, focal zone position, time-gain compensation, dynamic range, and transmit frequency were locked to study presets, and the operator placed the probe perpendicular to the breast surface with mild contact to avoid pre-compression that would bias the SWE measurement. For each examination, three orthogonal scans of the index lesion were obtained, and patients were positioned identically at the baseline, week 1, and week 3 visits. Image quality was reviewed in real time, and acquisitions with motion artifact, incomplete lesion coverage, or reverberation were repeated before the patient left the imaging suite. The locked acquisition presets comprised an imaging depth of 4 cm, a single focal zone positioned at the center of the index lesion, time-gain compensation held at the neutral mid-position, a dynamic range of 65 dB, a transmit frequency of 9 MHz for B-mode and radiofrequency capture, and a fixed SWE color-box scale; three orthogonal scans were obtained per lesion to average out plane-dependent sampling variability, and probe pre-compression was avoided because applied stress artificially raises measured stiffness and would bias the biomechanical features.

Region-of-interest segmentation was performed offline by a breast radiologist with more than ten years of experience and verified independently by a second reader; both readers were blinded to the pathologic outcome. The intratumoral region of interest enclosed the lesion margins identified on B-mode, while a 3 mm peritumoral ring was added because the peritumoral environment carries stromal and inflammatory information relevant to chemoresponse. From the radiofrequency-derived parametric maps, three spectral features (mid-band fit, spectral slope, and spectral intercept) and three texture features (entropy, homogeneity, and peritumoral heterogeneity) were computed for every lesion at every timepoint. From the SWE elastograms, mean stiffness, maximum stiffness, stiffness heterogeneity (standard deviation within the lesion), and a paired strain ratio (lesion versus surrounding fat reference) were extracted. All feature extraction was performed at the patient level to avoid fragment-based overrepresentation, consistent with the methodology used in previous multi-institutional QUS studies [20]. Region-of-interest segmentation was performed by an experienced breast radiologist and independently verified by a second blinded reader to ensure reproducibility, and a 3 mm peritumoral ring was appended to the intratumoral ROI because the immediately adjacent stroma carries inflammatory and desmoplastic information that changes early with effective therapy; feature extraction was aggregated at the patient level rather than per image fragment so that lesions contributing more fragments would not be over-represented [20].

Delta features were defined as week 1 minus baseline (Δ week 1) and week 3 minus baseline (Δ week 3) for every continuous variable, yielding 20 paired kinetic features per patient. Inter-reader agreement for segmentation and feature extraction was tested in a random subset of 30 patients using intraclass correlation coefficients (ICCs) and resulted in excellent agreement for all spectral and elastography parameters (all ICCs > 0.84). To control for acquisition drift, daily phantom measurements were performed on a tissue-mimicking gelatin block, and any week with phantom drift exceeding 5% of the reference value was flagged for review; no patient required exclusion on this basis. Early percentage tumor size change in ultrasound was calculated using the maximum diameter and the orthogonal diameter at week 3 relative to baseline. A reproducibility subsample of 30 patients was pre-specified for the inter-reader ICC analysis; for an expected ICC of approximately 0.85 with two readers this size yields a two-sided 95% confidence interval of roughly ±0.1, which is adequate to establish good-to-excellent agreement while limiting the burden of duplicate segmentation across the full cohort. Nonetheless, restricting the reproducibility analysis to 30 of the 135 patients is a limitation of the present work: although the subsample was adequately powered for the intended purpose, the resulting ICC estimates may not fully capture inter-reader variability across the entire range of lesion sizes, depths, and echogenicities encountered in the full cohort.

### 2.4. Statistical Analysis and Machine Learning Framework

Continuous variables were assessed for normality using the Shapiro–Wilk test and visual inspection of Q-Q plots. Normally distributed variables are reported as mean ± standard deviation and were compared using Welch’s *t*-test; skewed variables were summarized as median (interquartile range) and compared using the Mann–Whitney U test. Categorical variables are presented as counts and percentages and were compared using the chi-square test, with Fisher’s exact test substituted when more than 20% of expected cell counts were below 5. Correlations between continuous imaging variables and pathologic markers used Spearman’s rank correlation coefficient; point-biserial correlations were used for binary outcomes. Effect sizes are reported as Cohen’s d for continuous variables and odds ratios with 95% confidence intervals for categorical variables. A two-sided *p*-value of less than 0.05 was considered statistically significant; for the multiple-comparison sections, Benjamini–Hochberg false discovery rate adjustment was applied.

A stacked machine learning ensemble was developed to predict favorable pathologic response. The following four base learners were trained in parallel: extreme gradient boosting (XGBoost), random forest, support vector machine with radial basis kernel, and L2-penalized logistic regression. Their out-of-fold predicted probabilities were combined by a logistic regression meta-learner, with isotonic recalibration of the final score. Hyperparameters were tuned by nested 10-fold cross-validation with the inner loop used for tuning and the outer loop used for unbiased performance estimation. Model discrimination was summarized using the area under the receiver operating characteristic curve (AUC) with bootstrap 95% confidence intervals (1000 iterations), sensitivity, specificity, accuracy, positive predictive value, and negative predictive value. The architecture therefore comprised the following two stages: four first-level base learners and a single second-level logistic meta-learner (distinct from the L2-penalized logistic base learner). To prevent information leakage, the base-learner probabilities supplied to the meta-learner were generated strictly as out-of-fold predictions within the inner cross-validation loop and were not separately pre-calibrated; the isotonic recalibration was fitted only on inner training folds and applied to the corresponding held-out fold, never on the outer test folds. Continuous features were z-score-standardized using statistics estimated on training folds only, and no imputation was required because complete-case eligibility was enforced. Hyperparameters were tuned by grid search over pre-specified ranges (XGBoost: max_depth 2–6, learning rate 0.01–0.30, 100–600 trees; random forest: 200–800 trees, max features √p–p/2; SVM-RBF: C 0.1–100, γ 10^−4^–10^−1^; and logistic: L2 penalty C 0.01–10), with the final configurations selected by inner-loop AUC.

Model calibration was assessed using the Brier score, the Hosmer–Lemeshow test, and visual inspection of calibration curves. Incremental value of QUS and SWE features over the clinical-only baseline was quantified using the continuous net reclassification improvement (NRI) and the integrated discrimination improvement (IDI). Clinical utility was evaluated using decision curve analysis with net benefit reported at decision thresholds of 30%, 40%, 50%, and 60% [21]. AUC differences between models were tested using DeLong’s test for correlated curves. Feature importance and directionality were derived from SHAP values, computed on the held-out folds and aggregated across the ensemble. Bootstrap stability of the top-ranked features was estimated by repeating the entire training pipeline on 1000 resamples and reporting rank stability and the proportion of bootstraps in which each feature remained within the top ten. All analyses were performed in Python 3.11.5 using scikit-learn 1.3.0, xgboost 1.7.6, and shap 0.42.1.

### 2.5. Mathematical Formulation of the mpUS Features and Ensemble

For each region of interest, the normalized power spectrum P(f) was obtained by dividing the tumor backscatter spectrum by a reference phantom spectrum. A linear regression was fitted to P(f) in decibels over the analysis bandwidth [f1, f2]: P(f) ≈ m·f + b, where the spectral slope is m (dB/MHz) and the spectral intercept is b (dB); the mid-band fit (MBF, dB) is the value of the fitted line at the center frequency fc = (f1 + f2)/2, i.e., MBF = m·fc + b.

Texture features were computed from the gray-level co-occurrence matrix (GLCM) p(i, j), and the normalized frequency of gray-level pairs (i, j) at a fixed offset. Entropy = −Σi Σj p(i, j)·log p(i, j) increases with textural disorder, whereas homogeneity = Σi Σj p(i, j)/(1 + |i − j|) increases with local uniformity; peritumoral heterogeneity was the entropy computed within the 3 mm peritumoral ring.

Shear-wave elastography converts the shear-wave propagation speed cs (m/s) into Young’s modulus E under the assumptions of tissue incompressibility and local isotropy, E = 3ρcs^2^, where ρ ≈ 1000 kg/m^3^ is the tissue density; mean and maximum stiffness and the within-lesion standard deviation (stiffness heterogeneity) were derived from the E map, and the strain ratio was the lesion-to-fat elasticity ratio.

For every continuous feature x, kinetic (delta) features were defined as Δx_week1 = x_week1 − x_baseline and Δx_week3 = x_week3 − x_baseline, yielding 20 paired kinetic descriptors per patient.

In the stacked ensemble, each base learner k produced an out-of-fold probability z_k = f_k(x); the second-level logistic meta-learner combined these into the final probability y = σ(β0 + Σk βk·z_k), where σ is the logistic function, followed by isotonic recalibration g(·) so that the reported score was g(y). Feature attributions used Shapley values, φj = Σ(S ⊆ F\{j}) [|S|!(|F| − |S| − 1)!/|F|!]·[v(S ∪ {j}) − v(S)], where F is the full feature set and v(S) the model output using subset S; the mean absolute Shapley value across patients defined global feature importance.

## 3. Results

### 3.1. Cohort Overview

The final cohort comprised 135 women with stage II–III breast cancer treated with NAC at the Pius Brinzeu County Emergency Hospital between January 2024 and December 2025. Sixty patients (44.4%) achieved a favorable pathologic response (RCB 0/I) and 75 (55.6%) were classified as non-responders (RCB II/III). The mean age was 52.9 ± 9.0 years, the most frequent molecular subtype was Luminal B, followed by TNBC and HER2-positive disease, and approximately two-thirds of tumors presented at clinical stage II. Baseline characteristics and the distribution of imaging features were broadly representative of a real-world European breast oncology population [22] and supported the assumption that responders and non-responders were imaging-comparable at intake apart from a small number of biologically relevant differences. In summary, the final cohort comprised 135 patients, of whom 60 (44.4%) achieved a favorable pathologic response (RCB 0/I) and 75 (55.6%) did not, and the most common molecular subtype was Luminal B, followed by TNBC and HER2-positive disease. The baseline clinicopathologic characteristics of the two response groups are summarized in Table 1.

Age was numerically higher among responders (54.3 ± 9.2 vs. 51.7 ± 8.7 years), but this difference did not reach significance (*p* = 0.097) and is therefore reported as a non-significant trend rather than a between-group difference. Body mass index was nearly identical between groups (27.4 ± 4.3 vs. 27.8 ± 4.1 kg/m^2^, *p* = 0.582), arguing against an obesity-mediated confounder in the imaging analysis. Pretreatment tumor diameter was modestly smaller in responders, with a mean difference of 3.4 mm (95% CI −6.8 to −0.04, *p* = 0.049, Cohen’s d = −0.34), but this difference was small in absolute terms and unlikely to drive a clinically actionable separation on its own. The most pronounced biologic difference was the proliferative activity captured by Ki-67, which was substantially higher in responders (49.2 ± 14.3% vs. 40.7 ± 13.8%, *p* < 0.001, d = 0.61), consistent with the established observation that more proliferative tumors tend to be more chemosensitive in aggressive subtypes. Molecular subtype distribution differed significantly (*p* = 0.003), with Luminal B tumors overrepresented among non-responders (50.7% vs. 23.3%; OR 0.29, 95% CI 0.13–0.62 for favorable response) and TNBC overrepresented among responders (48.3% vs. 28.0%; OR 2.41, 95% CI 1.16–5.00). The corresponding baseline multi-parametric ultrasound features are presented in Table 2.

The spectral parameters mid-band fit, spectral slope, and spectral intercept did not separate responders and non-responders at the pretreatment timepoint (all *p* ≥ 0.157), suggesting that the average scatterer concentration and acoustic coupling were similar across tumors regardless of their eventual response. Mean and maximum SWE stiffness were also comparable between groups (mean stiffness 128.4 ± 29.4 vs. 124.8 ± 29.4 kPa, *p* = 0.480; maximum 182.7 ± 33.1 vs. 178.3 ± 31.6 kPa, *p* = 0.432), consistent with the observation that fully untreated invasive carcinomas tend to converge to a stiff, dense baseline regardless of chemosensitivity. In contrast, lower intratumoral homogeneity (0.3 ± 0.1 vs. 0.4 ± 0.1, *p* = 0.008, d = −1.00) and higher peritumoral heterogeneity (0.9 ± 0.1 vs. 0.8 ± 0.2, *p* = 0.034, d = 0.61) were both associated with eventual favorable response. SWE stiffness heterogeneity, defined as the standard deviation of stiffness values within the lesion ROI, was significantly higher in responders (47.2 ± 11.4 vs. 39.6 ± 10.7 kPa, *p* < 0.001, d = 0.69). The early kinetic changes recorded at week 1 and week 3 are summarized in Table 3.

The Δ MBF was approximately 2.4 times larger in responders at week 1 (+1.7 ± 0.6 vs. +0.7 ± 0.5 dB, d = 1.82) and approximately 2.8 times larger at week 3 (+3.4 ± 0.9 vs. +1.2 ± 0.8 dB, d = 2.59). The Δ entropy showed one of the strongest separations in the entire dataset, with a Cohen’s d of 3.00 at week 1 and 2.50 at week 3, indicating that the disorganization of the scatterer field after cytotoxic exposure is one of the most discriminative early-response phenomena. The SWE-derived features added a clearly independent biomechanical dimension as follows: Δ mean stiffness at week 1 reached d = −2.66 (−18.7 ± 5.4 vs. −5.2 ± 4.8 kPa), and at week 3 it climbed to d = −3.51 (−44.1 ± 9.7 vs. −12.1 ± 8.6 kPa), reflecting a far more aggressive softening of the tumor in chemosensitive patients. Importantly, the early ultrasound dimensional change at week 3 (−26.3 ± 7.4% vs. −11.7 ± 6.2%) was meaningful but smaller in standardized effect (d = −2.16) than the radiomic and elastography kinetics, supporting the hypothesis that microstructural and biomechanical change precedes and exceeds macroscopic shrinkage. The biologic credibility of these findings is reinforced by the much larger Ki-67 reduction (22.7 ± 7.6 vs. 9.4 ± 6.1 percentage points) and markedly lower residual cellularity (16.8 ± 8.4% vs. 43.9 ± 13.7%) at definitive surgery in responders. At week 1, Δ MBF was already about 2.4-fold larger in responders (+1.7 vs. +0.7 dB); Δ entropy was among the most discriminative kinetic biomarkers (Cohen’s d = 3.00 at week 1). The multivariable logistic regression model for favorable pathologic response is reported in Table 4.

The week 3 change in mid-band fit retained an adjusted odds ratio of 23.7 per 1 dB increase (95% CI 3.4–164.8, *p* = 0.002), and the week 3 change in entropy showed an adjusted OR of 2.91 per 0.1-unit increase (95% CI 1.62–5.23, *p* < 0.001). The biomechanical kinetic signal also survived adjustment as follows: a 10 kPa decrease in mean SWE stiffness at week 3 was associated with an adjusted OR of 1.87 (95% CI 1.18–2.96, *p* = 0.008). Among baseline variables, only intratumoral homogeneity retained independent significance, with each 0.1-unit increase in baseline homogeneity associated with 38% lower odds of favorable response (OR 0.62, 95% CI 0.39–0.98, *p* = 0.041). The predictive performance of the progressively richer models, including the stacked machine learning ensemble, is presented in Table 5.

The clinical-only model—including age, tumor size, Ki-67, subtype, and stage—achieved an AUC of 0.71 (95% CI 0.62–0.79) with a Brier score of 0.213, representing a useful but modest baseline. Adding baseline multi-parametric ultrasound features increased the AUC to 0.77 (95% CI 0.69–0.85) and decreased the Brier score to 0.184, confirming that pretreatment heterogeneity descriptors and SWE stiffness heterogeneity provide non-trivial information beyond clinical variables alone. The greatest performance gain emerged when the model incorporated kinetic information from week 1, with the AUC reaching 0.83 (95% CI 0.76–0.90); the subsequent jump to 0.89 with week 3 kinetics (95% CI 0.83–0.94) emphasized that the additional cycle of treatment carries meaningful incremental information beyond the very early week 1 signal. The stacked ML ensemble, which combined the outputs of XGBoost, random forest, support vector machine, and logistic regression via a logistic meta-learner and isotonic calibration, achieved an AUC of 0.93 (95% CI 0.88–0.97), accuracy of 88.9%, and well-balanced sensitivity (88.3%) and specificity (89.3%) on nested cross-validation. The Brier score of 0.083 indicates excellent calibration, with a positive predictive value of 86.9% and a negative predictive value of 90.5%. Across the ten outer cross-validation folds, the ensemble AUC was 0.93 ± 0.04 (range 0.86–0.98); this fold-level spread reflects the modest test-fold size (approximately 13 patients per fold) and should be considered when interpreting the point estimate. Across the modeling sequence, discrimination rose monotonically from an AUC of 0.71. The Spearman correlations between the multi-parametric ultrasound features and the pathologic markers of response are shown in Table 6.

The week 3 change in mid-band fit correlated negatively with residual cellularity at surgery (rho = −0.67, *p* < 0.001) and positively with the percentage point reduction in Ki-67 between baseline and week 3 (rho = +0.61, *p* < 0.001), indicating that the larger the scatterer-driven acoustic response, the smaller the residual disease burden and the more pronounced the proliferative collapse. The week 3 change in entropy showed an essentially parallel pattern (rho = −0.59 and rho = +0.54, respectively, both *p* < 0.001), confirming that the gain in microstructural disorder after effective cytotoxic exposure is biologically coherent with cellular and stromal turnover. The biomechanical features added an independent dimension as follows: the week 3 change in mean SWE stiffness correlated positively with residual cellularity (rho = +0.62, *p* < 0.001) and negatively with Ki-67 reduction (rho = −0.51, *p* < 0.001), consistent with stiffness loss being a hallmark of effective treatment. Baseline features correlated more weakly but in the expected direction, with baseline SWE heterogeneity showing the strongest baseline relationship (rho = −0.31, *p* < 0.001 with residual cellularity).

### 3.2. Subgroup and Interaction Analyses

In Luminal B disease, responders showed an average week 3 Δ MBF of +2.6 dB versus +1.1 dB in non-responders (Cohen’s d = 1.93) and a SWE stiffness decrease of −31.7 kPa versus −10.4 kPa (d = −2.61). The corresponding signals were noticeably larger in HER2-positive tumors (Δ MBF d = 2.83; Δ SWE d = −4.31) and largest in TNBC (Δ MBF d = 3.21; Δ SWE d = −4.62). Formal interaction testing using a linear model with interaction terms confirmed a significant modulation of the imaging response by subtype, with subtype × Δ MBF interaction *p* = 0.019 for TNBC and *p* = 0.043 for HER2-positive disease when compared to Luminal B as the reference. The subtype-specific AUC of the full stacked ensemble was 0.87 (95% CI 0.78–0.94) in Luminal B, 0.94 (0.85–0.99) in HER2-positive, and 0.95 (0.87–0.99) in TNBC. Because the per-subtype samples are small (Luminal B *n* = 52, HER2-positive *n* = 33, TNBC *n* = 50), the subtype-specific AUC values (0.87–0.95) are statistically unstable and should be regarded as exploratory and hypothesis-generating rather than confirmatory. The subtype-specific magnitude of early mpUS change, together with the formal interaction testing and the subtype-specific model performance, is detailed in Table 7.

The SHAP-based ranking of feature importance, together with the corresponding bootstrap stability estimates, is presented in Table 8.

The three most influential features were all week 3 kinetic variables as follows: Δ MBF (mean |SHAP| = 0.71, 95% CI 0.62–0.79), Δ entropy (0.62, 95% CI 0.54–0.71) and Δ SWE mean stiffness (0.54, 95% CI 0.46–0.62). Crucially, these features were also remarkably stable across bootstrap resamples, with rank stability indices of 0.94, 0.91, and 0.89, respectively, and with each feature included in the top ten of more than 98% of bootstrap iterations. The week 1 kinetic counterparts (Δ MBF and Δ entropy at week 1) appeared in the next tier of importance, demonstrating that even the earliest treatment effect carries reliable predictive signal. Baseline features such as peritumoral heterogeneity, Ki-67, and homogeneity contributed measurable but considerably smaller and less stable SHAP magnitudes, with rank stability indices between 0.43 and 0.71 and top-ten inclusion rates between 57% and 89%. The molecular subtype TNBC indicator contributed an interpretable but modest SHAP impact (0.16, rank stability 0.49), consistent with the multivariable analysis in Table 4. The decision curve analysis, the DeLong AUC comparisons, and the reclassification statistics relative to the clinical-only model are reported in Table 9.

Adding baseline multi-parametric ultrasound to the clinical model produced a statistically significant AUC gain of 0.06 (DeLong *p* = 0.041), a continuous NRI of +0.213, and an IDI of +0.064, demonstrating that even pretreatment imaging features add genuine reclassification value rather than only curve-fitting. The introduction of week 1 kinetic features improved the AUC by 0.12 versus the clinical baseline (DeLong *p* = 0.003), with NRI rising to +0.387 and IDI to +0.118. Adding week 3 kinetics further increased the AUC gain to 0.18 (DeLong *p* < 0.001) and produced a continuous NRI of +0.524 and IDI of +0.171, both indicating substantial improvement in the separation of predicted probabilities between responders and non-responders. The stacked ensemble combining all features delivered the largest improvement, with an AUC gain of 0.22 versus clinical alone (DeLong *p* < 0.001), an NRI of +0.611, and an IDI of +0.219. Decision curve analysis showed that this advantage translated into consistently higher net clinical benefit across the relevant decision thresholds as follows: at a 30% probability threshold, the net benefit of the stacked ensemble (0.346) was approximately double that of the clinical-only model (0.171), and the advantage was preserved at 50% (0.281 vs. 0.118) and 60% (0.224 vs. 0.087).

**Figure 1:**
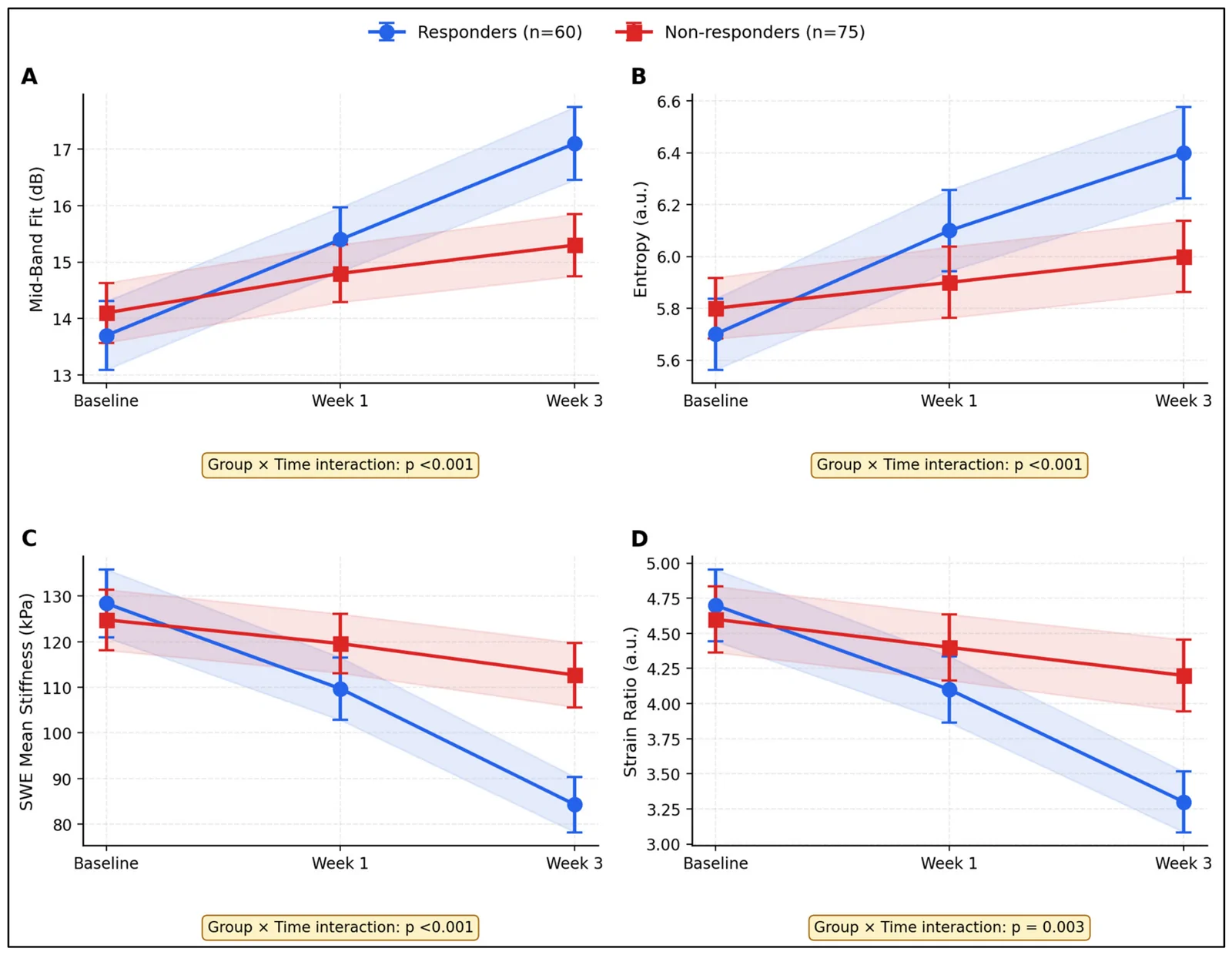
(**A**–**D**) Kinetic trajectories of four representative multi-parametric ultrasound features in responders (*n* = 60) and non-responders (*n* = 75) across baseline, week 1, and week 3. Error bars represent 95% confidence intervals. Group × Time interaction *p*-values were derived from a linear mixed-effect model with patient-level random intercepts.

Figure 1 displays the multi-timepoint kinetic trajectories of four mpUS features that diverged most sharply between response groups. Panel A shows that mid-band fit increased progressively in responders (13.7 → 15.4 → 17.1 dB) while non-responders changed minimally (14.1 → 14.8 → 15.3 dB), producing a Group × Time interaction *p* < 0.001. Panel B shows that entropy followed a parallel pattern (responders 5.7 → 6.1 → 6.4; non-responders 5.8 → 5.9 → 6.0; interaction *p* < 0.001). Panel C shows the SWE-derived softening as follows: responders dropped from 128.4 to 84.3 kPa in three weeks, a 34.3% reduction, while non-responders softened by only 9.7% (124.8 → 112.7 kPa), with interaction *p* < 0.001. Panel D shows that the strain ratio decreased steeply in responders (4.7 → 4.1 → 3.3) but only marginally in non-responders (4.6 → 4.4 → 4.2), with interaction *p* = 0.003.

**Figure 2:**
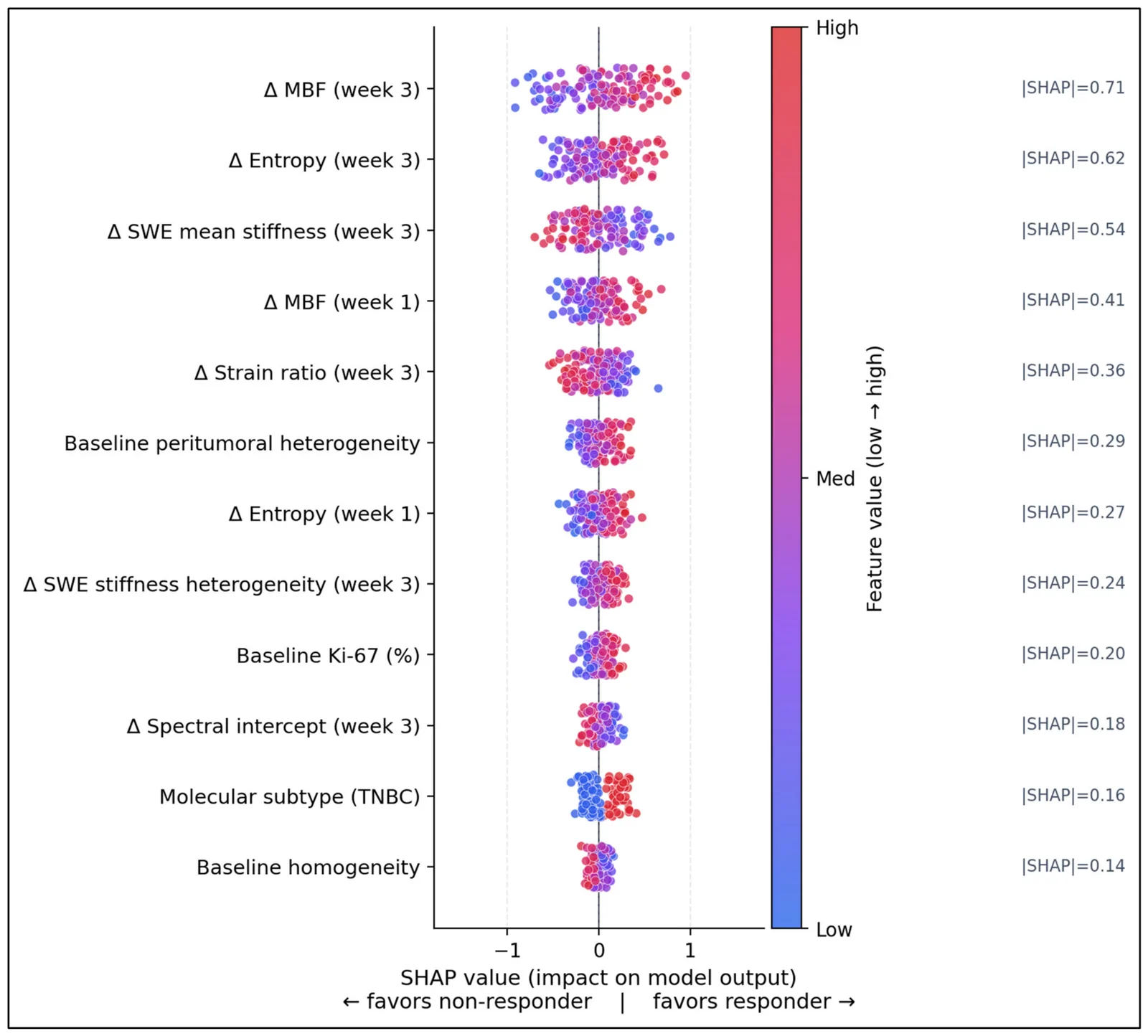
SHAP beeswarm plot showing the per-patient contribution of the top 12 features to the stacked machine learning ensemble. Each point represents one patient. The x-axis shows the SHAP value (impact on the model output, with positive values favoring a responder prediction); the y-axis ranks the features by mean absolute SHAP magnitude. Color encodes the feature value (low to high). For each feature row, the number of plotted points equals the full cohort (*n* = 135 patients).

Figure 2 makes the internal logic of the stacked ML ensemble explicit at the patient level. The week 3 change in mid-band fit (mean |SHAP| = 0.71) dominated the predictions, with high values pushing the model strongly toward a responder classification and low values pulling it toward non-responder. The week 3 changes in entropy (|SHAP| = 0.62) and SWE mean stiffness (|SHAP| = 0.54) acted in the expected biologic direction—greater entropy increase favored responder classification, while less stiffness reduction (high color value) pushed predictions toward non-responder. Week 1 features contributed earlier but smaller weights (Δ MBF |SHAP| = 0.41; Δ entropy |SHAP| = 0.27), consistent with their role as early indicators. The baseline peritumoral heterogeneity (|SHAP| = 0.29) acted as a pretreatment modifier: tumors with high baseline peritumoral disorder were nudged toward responder predictions even before any kinetic signal was integrated. The TNBC indicator (|SHAP| = 0.16) and baseline Ki-67 (|SHAP| = 0.20) also contributed coherently.

**Figure 3:**
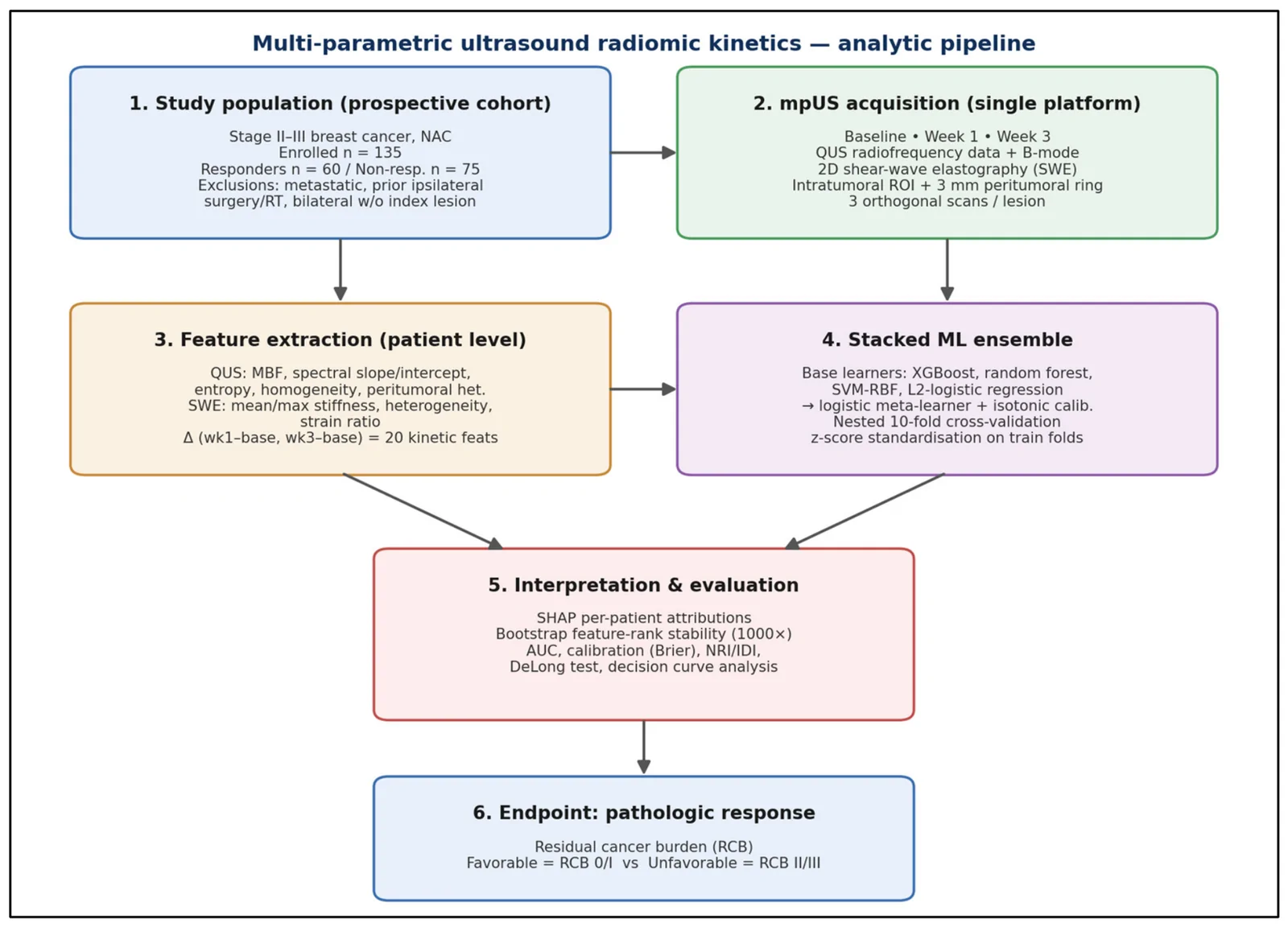
Overview of the study workflow and analytic pipeline, from prospective enrollment and standardized multi-parametric ultrasound acquisition at baseline, week 1, and week 3, through patient-level QUS and SWE feature extraction and kinetic (Δ) feature construction, to the stacked machine-learning ensemble, SHAP interpretation with bootstrap stability assessment, and the pathologic response endpoint (RCB 0/I vs. II/III).

**Figure 4:**
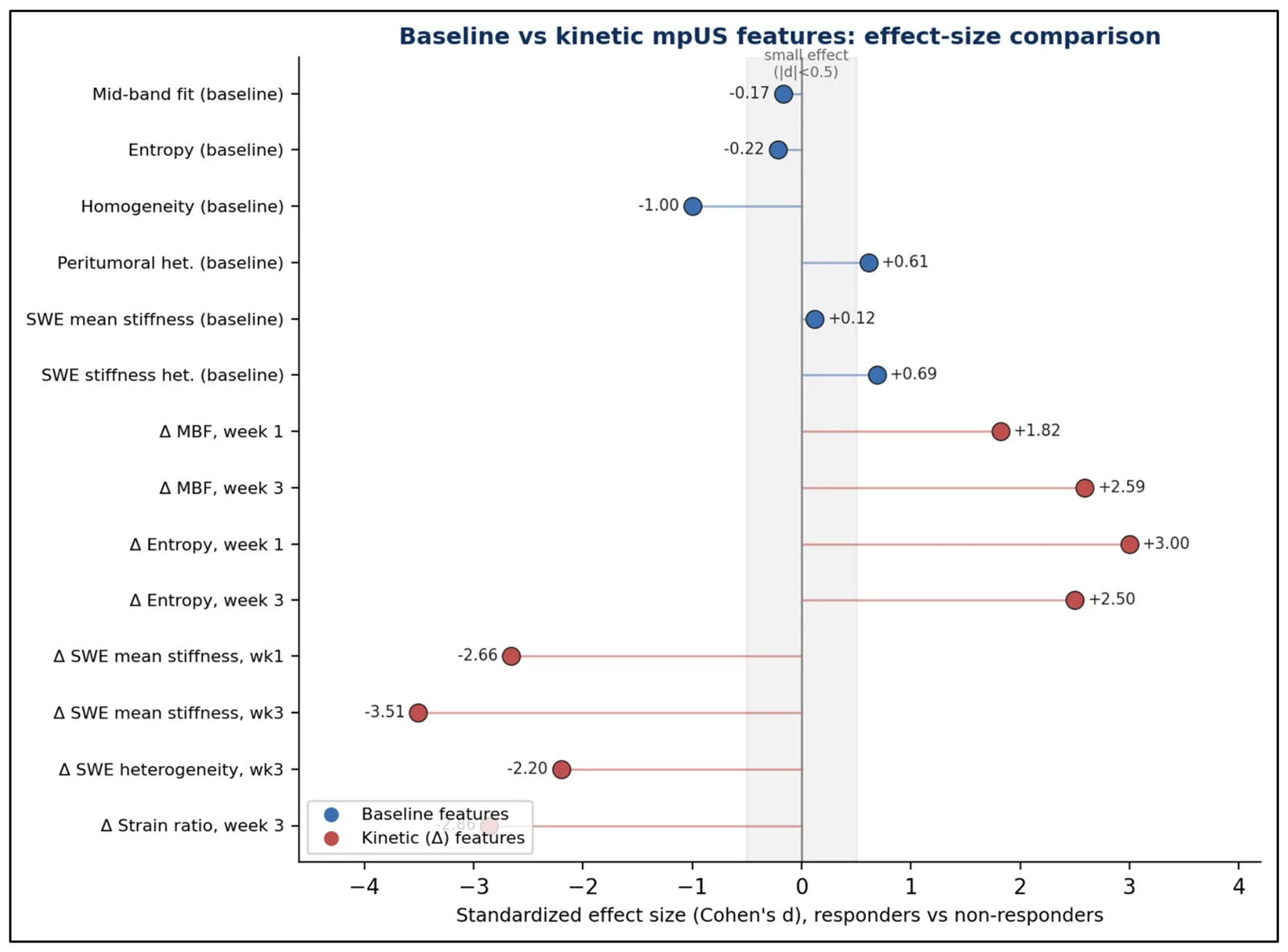
Forest plot of standardized effect sizes (Cohen’s d, responders vs. non-responders) for baseline multi-parametric ultrasound features (Table 2) and early kinetic (Δ) features (Table 3). Baseline features cluster near the null (shaded |d| < 0.5 region), whereas the week 1 and week 3 kinetic features show large effect sizes, making the contrast between non-informative baseline descriptors and highly discriminative kinetic descriptors visually immediate.

Figure 3 summarizes the overall study workflow and analytic pipeline, from prospective enrollment and standardized multi-parametric ultrasound acquisition to the stacked machine learning ensemble and the pathologic response endpoint, and Figure 4 contrasts the standardized effect sizes of the baseline features with those of the week 1 and week 3 kinetic features.

## 4. Discussion

This study suggests that combining quantitative ultrasound radiomics with shear wave elastography in a kinetic, multi-timepoint framework can provide an early, biologically coherent prediction of pathologic response to neoadjuvant chemotherapy in breast cancer. The baseline analysis revealed that tumors with lower intratumoral homogeneity, higher peritumoral heterogeneity, and higher SWE stiffness heterogeneity were more likely to eventually respond, while mean stiffness and mean scatterer intensity were not informative at the pretreatment stage [23,24]. The fact that homogeneity remained an independent predictor after multivariable adjustment further reinforces this notion and aligns with prior reports that texture-derivative QUS features can capture chemosensitivity signatures even before treatment begins [25]. Similar patterns have been described in more recent validation work demonstrating that pretreatment microstructural disorder is associated with downstream pathologic response [26], and pretreatment ultrasound-derived deep learning radiomic models have likewise highlighted the importance of microstructural information in early prediction [27]. Mean stiffness and mean scatterer intensity were uninformative at baseline because untreated carcinomas converge to a uniformly stiff, densely packed state, so absolute values carry little discriminative signal; the discriminative information instead resided in disorder-based descriptors and, above all, in their on-treatment kinetics.

The strongest novel contribution of this work is the demonstration that mpUS kinetic features outperform baseline imaging, clinical biology, and even single-modality QUS or SWE assessments when integrated into a stacked machine learning ensemble. The week 3 changes in mid-band fit, entropy, and SWE mean stiffness produced very large effect sizes (Cohen’s d 2.5–3.5), correlated strongly with both Ki-67 reduction and residual cellularity, and remained independently associated with response after adjustment, consistent with previous reports demonstrating that delta-radiomic features track treatment efficacy better than baseline-only descriptors [28]. Earlier QUS work has shown that the change in spectral and textural parameters captured during the first cycles of NAC is associated with both pathologic response and recurrence-free survival [29], and multimodal ultrasound combinations have recently extended this observation in larger cohorts [30]. The biomechanical kinetic signal observed in our SWE features is also in line with earlier evidence that elastographic softening is predictive of response [31], and direct comparisons between strain and shear-wave elastography have suggested a complementary role for the two techniques [32]. Delta-radiomic features outperform baseline-only descriptors for treatment monitoring because they measure the change a specific tumor undergoes under a specific regimen—an inherently patient- and treatment-specific readout—rather than a static pretreatment property, and this on-treatment change is what the pathologic endpoint ultimately reflects.

From a clinical translation perspective, mpUS-based monitoring is attractive because it is contrast-free, radiation-free, repeatable, and considerably less expensive than MRI, building on the established performance of shear-wave elastography for breast lesion characterization in large multinational cohorts [33]. The fact that week 1 features were already informative is particularly important because it raises the possibility of intervening before substantial cytotoxic exposure to an ineffective regimen has occurred, in line with broader perspectives on the future direction of locally advanced breast cancer management [34]. Subtype-specific interaction analyses, however, showed that the magnitude of the kinetic response varied across Luminal B, HER2-positive, and TNBC tumors. This suggests that subtype-specific calibration will be necessary before deployment, with comparisons between models supported by formal statistical methodology for correlated AUCs [35], reclassification metrics that quantify added predictive value [36], and contemporary frameworks for the comprehensive assessment of prediction models [37]. mpUS-based monitoring is attractive for clinical translation relative to MRI because it is contrast-free, radiation-free, inexpensive, and repeatable at the bedside within a single visit, which makes weekly reassessment feasible; the subtype-dependence of the kinetic response, however, means that decision thresholds will require subtype-specific calibration and harmonized acquisition protocols before deployment, an effort that parallels broader work on cross-dataset harmonization for imaging-based breast cancer models [38] and the curation of harmonized, expert-annotated mammographic datasets assembled specifically to support generalizable artificial-intelligence models across institutions and populations [39].

The principal novelty and contribution of this work are threefold. First, to our knowledge it is the first study to fuse QUS spectral–textural radiomics with SWE elasticity into a single multi-timepoint kinetic signature rather than treating the two modalities, or the individual timepoints, in isolation. Second, it embeds these kinetic features in a stacked ensemble with SHAP interpretability and bootstrap-based stability assessment, so that the model is both accurate and transparent at the level of the individual patient. Third, it demonstrates that the early on-treatment kinetics (weeks 1–3) subsume much of the predictive value of conventional pretreatment biology, quantifies the subtype-dependence of this signal, and benchmarks the approach against recent ultrasound-radiomic studies (Table 10). The specific contribution of each author to these components is detailed in the Author Contributions section.

Table 10 situates the present work among recent ultrasound-radiomic studies. Reported discrimination is broadly comparable (AUC ~0.89–0.94), but most prior models rely on a single ultrasound modality and one or two timepoints and are validated internally; the distinguishing features of the present study are the combination of QUS and SWE, the explicit multi-timepoint kinetic modeling, and the interpretable stacked ensemble. As with those studies, all performance figures reported here reflect internal validation, and external multicenter testing remains necessary.

## 5. Study Limitations

Several limitations should temper the interpretation of these results. First, the study was conducted at a single center, with imaging performed on a single platform by two operators trained with a shared protocol; while this design minimized technical heterogeneity, it inherently limits generalizability to other scanners, transducers, and signal-processing pipelines. Second, although 135 patients are a meaningful prospective cohort for mpUS work, the sample size is modest for a high-dimensional radiomic and machine learning analysis, particularly when subtype-stratified analyses are considered. Inter-reader reproducibility was likewise established in a pre-specified subsample of 30 patients rather than in the complete cohort, so the reported ICCs carry a correspondingly wider confidence interval than a full-cohort analysis would have produced. The wide confidence interval around the Δ MBF multivariable odds ratio reflects this limitation. Third, the pathologic response endpoint was dichotomized at RCB class I/II, which is clinically sensible but simplifies a biologically continuous process. Fourth, the analysis did not include genomic or transcriptomic data, which could in principle refine the multi-parametric model further. Fifth, no external validation cohort was available, so the reported AUC values are likely optimistic relative to true out-of-sample performance. Finally, the model was not designed for metastatic disease, pregnancy, prior treated breast tumors, or lesions inconsistently visualized on ultrasound, and any future deployment will require subtype-specific calibration and harmonized acquisition protocols. Using a single ultrasound platform minimized technical heterogeneity but limits generalizability, and 135 patients—though a substantial prospective mpUS cohort—remain modest for high-dimensional radiomic and machine learning analysis, particularly within subtype strata, which is why the reported AUCs (internal validation only) are likely optimistic relative to true out-of-sample performance; the model was not developed for metastatic, pregnant, previously treated, or sonographically occult tumors, and incorporating genomic or transcriptomic data could further refine prediction, so external multicenter validation with harmonized, subtype-specific calibration is the necessary next step [38].

## 6. Conclusions

Multi-parametric ultrasound radiomic kinetics, acquired across the early weeks of neoadjuvant chemotherapy and integrated through an interpretable stacked machine learning ensemble, showed strong potential for early prediction of pathologic response in breast cancer. The early on-treatment changes in scatterer-based radiomic features and shear-wave stiffness were the most informative and most reproducible predictors, and the combined clinical-plus-multiparametric ultrasound model outperformed clinical, baseline-imaging, and single-modality approaches, with the most pronounced response signal in the more aggressive subtypes. These findings indicate that response to cytotoxic therapy can be detected at the tissue-microstructure and biomechanical level before macroscopic tumor shrinkage, and that interpretable machine learning can translate this signal into a clinically usable decision-support tool. Prospective multicenter validation, acquisition harmonization, and subtype-specific calibration remain necessary before clinical translation.

## Figures and Tables

**Table 1:** Baseline clinicopathologic characteristics according to pathologic response.

Variable	Responders (*n* = 60)	Non-Responders (*n* = 75)	Effect Estimate (95% CI)	*p*-Value
Age (years), mean ± SD	54.3 ± 9.2	51.7 ± 8.7	MD +2.6 (−0.5 to +5.7); d = 0.29	0.097
BMI (kg/m^2^), mean ± SD	27.4 ± 4.3	27.8 ± 4.1	MD −0.4 (−1.8 to +1.0); d = −0.10	0.582
Tumor size (mm), mean ± SD	38.7 ± 9.3	42.1 ± 10.6	MD −3.4 (−6.8 to −0.04); d = −0.34	0.049
Ki-67 (%), mean ± SD	49.2 ± 14.3	40.7 ± 13.8	MD +8.5 (+3.7 to +13.3); d = 0.61	<0.001
Histologic grade 3, *n* (%)	43 (71.7%)	42 (56.0%)	OR 2.01 (0.97–4.18)	0.061
Molecular subtype				0.003
Luminal B, *n* (%)	14 (23.3%)	38 (50.7%)	OR 0.29 (0.13–0.62)	—
HER2-positive, *n* (%)	17 (28.3%)	16 (21.3%)	OR 1.46 (0.67–3.18)	—
TNBC, *n* (%)	29 (48.3%)	21 (28.0%)	OR 2.41 (1.16–5.00)	—
Clinical stage III, *n* (%)	22 (36.7%)	26 (34.7%)	OR 1.09 (0.53–2.23)	0.806

For molecular subtype, the single *p*-value (0.003) is the omnibus χ^2^ test across all three categories; the category-specific odds ratios beneath it compare each subtype against the remainder for a favorable response and do not carry separate *p*-values. All other rows report a single variable-level comparison.

**Table 2:** Baseline multi-parametric ultrasound features.

Feature	Responders (*n* = 60)	Non-Responders (*n* = 75)	Effect Estimate (95% CI)	*p*-Value
Mid-band fit (dB)	13.7 ± 2.4	14.1 ± 2.3	MD −0.4 (−1.2 to +0.4); d = −0.17	0.328
Spectral slope (dB/MHz)	−0.7 ± 0.3	−0.6 ± 0.3	MD −0.1 (−0.21 to +0.04); d = −0.33	0.157
Spectral intercept (dB)	23.4 ± 4.2	22.8 ± 3.9	MD +0.6 (−0.8 to +2.0); d = 0.15	0.391
Entropy	5.7 ± 0.5	5.8 ± 0.4	MD −0.1 (−0.26 to +0.05); d = −0.22	0.183
Homogeneity	0.3 ± 0.1	0.4 ± 0.1	MD −0.1 (−0.14 to −0.04); d = −1.00	0.008
Peritumoral heterogeneity	0.9 ± 0.1	0.8 ± 0.2	MD +0.1 (+0.04 to +0.16); d = 0.61	0.034
SWE mean stiffness (kPa)	128.4 ± 29.4	124.8 ± 29.4	MD +3.6 (−6.4 to +13.6); d = 0.12	0.480
SWE maximum stiffness (kPa)	182.7 ± 33.1	178.3 ± 31.6	MD +4.4 (−6.6 to +15.4); d = 0.14	0.432
SWE stiffness heterogeneity (kPa)	47.2 ± 11.4	39.6 ± 10.7	MD +7.6 (+3.8 to +11.4); d = 0.69	<0.001
Strain ratio	4.7 ± 1.0	4.6 ± 1.0	MD +0.1 (−0.24 to +0.44); d = 0.10	0.567

*p*-values were adjusted for multiple comparisons using the Benjamini–Hochberg false discovery rate. After correction, homogeneity, peritumoral heterogeneity, and SWE stiffness heterogeneity remained significant (adjusted *p* < 0.05); the remaining baseline comparisons were non-significant before and after adjustment.

**Table 3:** Early kinetic changes at week 1 and week 3 according to pathologic response.

Δ Feature	Responders (*n* = 60)	Non-Responders (*n* = 75)	Effect Estimate (95% CI)	*p*-Value
Δ MBF, week 1 (dB)	+1.7 ± 0.6	+0.7 ± 0.5	MD +1.0 (+0.81 to +1.19); d = 1.82	<0.001
Δ MBF, week 3 (dB)	+3.4 ± 0.9	+1.2 ± 0.8	MD +2.2 (+1.91 to +2.49); d = 2.59	<0.001
Δ Entropy, week 1	+0.4 ± 0.1	+0.1 ± 0.1	MD +0.3 (+0.27 to +0.33); d = 3.00	<0.001
Δ Entropy, week 3	+0.7 ± 0.2	+0.2 ± 0.2	MD +0.5 (+0.43 to +0.57); d = 2.50	<0.001
Δ Spectral intercept, week 3 (dB)	−3.7 ± 1.3	−1.4 ± 1.2	MD −2.3 (−2.73 to −1.87); d = −1.83	<0.001
Δ SWE mean stiffness, week 1 (kPa)	−18.7 ± 5.4	−5.2 ± 4.8	MD −13.5 (−15.24 to −11.76); d = −2.66	<0.001
Δ SWE mean stiffness, week 3 (kPa)	−44.1 ± 9.7	−12.1 ± 8.6	MD −32.0 (−35.13 to −28.87); d = −3.51	<0.001
Δ SWE heterogeneity, week 3 (kPa)	−18.4 ± 5.7	−6.8 ± 4.9	MD −11.6 (−13.42 to −9.78); d = −2.20	<0.001
Δ Strain ratio, week 3	−1.4 ± 0.4	−0.4 ± 0.3	MD −1.0 (−1.12 to −0.88); d = −2.86	<0.001
Tumor size change, week 3 (%)	−26.3 ± 7.4	−11.7 ± 6.2	MD −14.6 (−16.94 to −12.26); d = −2.16	<0.001
Ki-67 reduction, week 3 (% points)	+22.7 ± 7.6	+9.4 ± 6.1	MD +13.3 (+10.92 to +15.68); d = 1.94	<0.001
Residual cellularity at surgery (%)	16.8 ± 8.4	43.9 ± 13.7	MD −27.1 (−31.05 to −23.15); d = −2.32	<0.001

**Table 4:** Multivariable logistic regression for favorable pathologic response.

Predictor	Adjusted OR	95% CI	Standardized β	*p*-Value
Age (per 5 years)	1.13	0.84–1.52	+0.12	0.412
Tumor size (per 10 mm)	0.71	0.43–1.17	−0.34	0.181
Ki-67 (per 10%)	1.42	0.93–2.16	+0.35	0.103
Molecular subtype: TNBC vs. Luminal B	2.81	0.94–8.39	+1.03	0.064
Molecular subtype: HER2+ vs. Luminal B	1.91	0.67–5.43	+0.65	0.226
Baseline SWE heterogeneity (per 5 kPa)	1.41	0.98–2.03	+0.34	0.066
Baseline homogeneity (per 0.1)	0.62	0.39–0.98	−0.48	0.041
Δ MBF, week 3 (per 1 dB)	23.7	3.4–164.8	+1.95	0.002
Δ Entropy, week 3 (per 0.1)	2.91	1.62–5.23	+1.07	<0.001
Δ SWE mean stiffness, week 3 (per −10 kPa)	1.87	1.18–2.96	+0.63	0.008

Footnote: for Δ MBF the odds ratio is expressed per 1 dB increase in the week 3 change in mid-band fit (reference unit = 1 dB); given the observed responder/non-responder difference of ~2.2 dB, a 1 dB increment represents slightly under half of the typical between-group separation. The wide confidence interval reflects near-complete separation and indicates an unstable estimate (see Section 4).

**Table 5:** Predictive performance of progressively richer models, including the stacked machine learning ensemble.

Model	AUC (95% CI)	Sensitivity	Specificity	Accuracy	PPV	NPV	Brier
Clinical only	0.71 (0.62–0.79)	64.4%	68.4%	66.7%	62.1%	70.6%	0.213
Clinical + baseline mpUS	0.77 (0.69–0.85)	71.7%	73.3%	72.6%	68.3%	76.4%	0.184
Clinical + Δ week 1 mpUS	0.83 (0.76–0.90)	78.3%	78.7%	78.5%	74.6%	81.9%	0.146
Clinical + Δ week 3 mpUS	0.89 (0.83–0.94)	83.3%	84.0%	83.7%	80.6%	86.3%	0.108
Stacked ML ensemble (all features)	0.93 (0.88–0.97)	88.3%	89.3%	88.9%	86.9%	90.5%	0.083

Footnote: all five rows use the identical stacked machine learning ensemble architecture (four base learners with a logistic meta-learner) and the same nested 10-fold cross-validation scheme; rows differ only in the feature set supplied to the ensemble, so incremental AUC gains reflect added feature information rather than changes in model complexity.

**Table 6:** Spearman correlations between multi-parametric ultrasound features and pathologic markers of response.

Imaging Feature	Residual Cellularity (Rho, *p*)	Ki-67 Reduction (Rho, *p*)	Tumor Size Change (Rho, *p*)
Baseline homogeneity	+0.27 (0.002)	−0.21 (0.014)	+0.18 (0.037)
Baseline peritumoral heterogeneity	−0.24 (0.005)	+0.19 (0.027)	−0.16 (0.063)
Baseline SWE heterogeneity (kPa)	−0.31 (<0.001)	+0.27 (0.002)	−0.22 (0.011)
Δ MBF, week 3 (dB)	−0.67 (<0.001)	+0.61 (<0.001)	−0.53 (<0.001)
Δ Entropy, week 3	−0.59 (<0.001)	+0.54 (<0.001)	−0.47 (<0.001)
Δ Spectral intercept, week 3 (dB)	+0.46 (<0.001)	−0.39 (<0.001)	+0.34 (<0.001)
Δ SWE mean stiffness, week 3 (kPa)	+0.62 (<0.001)	−0.51 (<0.001)	+0.49 (<0.001)
Δ SWE heterogeneity, week 3 (kPa)	+0.43 (<0.001)	−0.36 (<0.001)	+0.31 (<0.001)
Δ Strain ratio, week 3	+0.41 (<0.001)	−0.34 (<0.001)	+0.29 (0.001)

**Table 7:** Subtype-specific magnitude of early mpUS change with formal interaction testing and model performance.

Subtype (*n*)	Δ MBF, Week 3 (Resp vs. Non-Resp; d)	Δ SWE, Week 3 (Resp vs. Non-Resp; d)	Subtype-Specific AUC (95% CI)	Subtype × Δ Interaction p
Luminal B (*n* = 52)	+2.6 vs. +1.1; d = 1.93	−31.7 vs. −10.4; d = −2.61	0.87 (0.78–0.94)	ref
HER2-positive (*n* = 33)	+3.6 vs. +1.4; d = 2.83	−47.4 vs. −13.2; d = −4.31	0.94 (0.85–0.99)	0.043
TNBC (*n* = 50)	+3.9 vs. +1.3; d = 3.21	−51.2 vs. −13.8; d = −4.62	0.95 (0.87–0.99)	0.019
Pooled cohort (*n* = 135)	+3.4 vs. +1.2; d = 2.59	−44.1 vs. −12.1; d = −3.51	0.93 (0.88–0.97)	—

**Table 8:** SHAP-based feature importance ranking with bootstrap stability (1000 iterations).

Feature	Mean |SHAP|	Bootstrap 95% CI	Mean Rank (Rank Stability)	Top-10 Inclusion (%)
Δ MBF, week 3	0.71	0.62–0.79	1.3 (0.94)	99.7%
Δ Entropy, week 3	0.62	0.54–0.71	2.1 (0.91)	99.1%
Δ SWE mean stiffness, week 3	0.54	0.46–0.62	3.2 (0.89)	98.4%
Δ MBF, week 1	0.41	0.33–0.49	4.3 (0.83)	96.2%
Δ Strain ratio, week 3	0.36	0.28–0.43	5.4 (0.76)	93.7%
Baseline peritumoral heterogeneity	0.29	0.23–0.36	6.7 (0.71)	89.3%
Δ Entropy, week 1	0.27	0.21–0.34	7.1 (0.68)	87.6%
Δ SWE heterogeneity, week 3	0.24	0.18–0.31	7.9 (0.64)	84.1%
Baseline Ki-67 (%)	0.20	0.14–0.26	9.3 (0.59)	76.4%
Δ Spectral intercept, week 3	0.18	0.13–0.24	10.1 (0.54)	71.2%
Molecular subtype (TNBC indicator)	0.16	0.11–0.22	11.4 (0.49)	63.8%
Baseline homogeneity	0.14	0.09–0.19	12.6 (0.43)	57.1%

**Table 9:** Decision curve analysis, DeLong AUC comparisons, and reclassification statistics versus the clinical-only model.

Model	ΔAUC vs. Clinical (DeLong *p*)	Continuous NRI	IDI	Net Benefit @ 30%	Net Benefit @ 50%	Net Benefit @ 60%
Clinical only	ref	ref	ref	0.171	0.118	0.087
Clinical + baseline mpUS	+0.06 (0.041)	+0.213	+0.064	0.213	0.156	0.114
Clinical + Δ week 1 mpUS	+0.12 (0.003)	+0.387	+0.118	0.267	0.198	0.149
Clinical + Δ week 3 mpUS	+0.18 (<0.001)	+0.524	+0.171	0.318	0.247	0.193
Stacked ML ensemble (all features)	+0.22 (<0.001)	+0.611	+0.219	0.346	0.281	0.224

**Table 10:** Comparison of the present study with recent ultrasound-based radiomic/machine learning studies for predicting response to neoadjuvant chemotherapy in breast cancer (2024–2026).

Study (Year) [Ref]	US Modality	Timepoints (Kinetics)	ML/Analysis	Endpoint	Validation	Reported AUC
Wang et al., 2024 [40]	B-mode US radiomics + deep learning + clinical	Pretreatment (pre-NAC)	DLRC (radiomics + DL + logistic)	pCR	Internal (train/validation split)	0.914–0.937
Yao et al., 2025 [41]	B-mode US radiomics (intra- + peritumoral)	Pretreatment	Random forest (+ LDA/SVM/LR/AdaBoost)	pCR	Internal (train/test split)	0.931 (intratumoral)
Wan et al., 2025 [30]	Multimodal US radiomics + clinical	Pre-NAC + after 2 cycles	Clinical–radiomics model	pCR	Internal + prospective test	as reported †
Moore-Palhares et al., 2025 [26]	QUS texture (RF data)	Serial, on-treatment	QUS texture model	Response (early)	Prospective serial (internal)	as reported †
Putin et al., 2026 [18]	QUS radiomics (B-mode/RF)	Baseline + week 2	ML classifiers (QUS only)	RCB 0/I vs. II/III	Internal (single center)	as reported †
Putin et al., 2026 [10]	QUS radiomics (systematic review)	Various	Various ML	Response/pCR	Review of internal-validation studies	AUCs across studies †
This study, 2026	mpUS = QUS + SWE kinetics	Baseline + week 1 + week 3 (kinetic)	Stacked ensemble + SHAP	RCB 0/I vs. II/III	Internal (nested 10-fold CV)	0.93 (0.88–0.97)

† Performance figures are as reported in each source and are not directly comparable owing to differing endpoints (pCR vs. RCB), cohorts, and validation schemes; all are internal-validation estimates. Where a single AUC is not tabulated, the value is available in the cited source.

## Data Availability

The data presented in this study are available on reasonable request from the corresponding author. The data are not publicly available due to privacy and ethical restrictions.
